# Laparoscopic Sigmoid Colectomy with Natural Orifice Specimen Extraction in Sigmoid Volvulus

**DOI:** 10.5152/eurasianjmed.2024.24420

**Published:** 2024-06-01

**Authors:** Sabri Selçuk Atamanalp

**Affiliations:** Department of General Surgery, Ataturk University Faculty of Medicine, Erzurum, Türkiye

**Keywords:** Sigmoid volvulus, elective treatment, laparoscopic sigmoid colectomy, natural orifice specimen extraction

## Abstract

Sigmoid volvulus (SV), the twisting of the sigmoid colon around its own base, is a relatively rare colonic obstruction form. Endoscopic detorsion is the first-line management option in uncomplicated patients. However, recurrence risk is as high as 90%, with a risk of mortality up to 35%. Although procedures such as sigmoidopexy, sigmoidomesopexy, sigmoidomesoplasty, extraperitonealization, or percutaneous endoscopic sigmoidopexy may prevent or reduce SV recurrence, laparoscopic sigmoid colectomy with natural orifice specimen extraction appears to be the optimal choice in selected cases.

**Main Points**

- Endoscopic detorsion is the first-line treatment route in uncomplicated patients with sigmoid volvulus (SV).
- However, SV tends to recur in 15%-55% of the patients following endoscopic detorsion, with a high mortality risk up to 35%.
- Although some procedures including sigmoidopexy, sigmoidomesopexy, sigmoidomesoplasty, extraperitonealization, or percutaneous endoscopic sigmoidopexy may prevent or reduce SV recurrence, laparoscopic sigmoid colectomy with natural orifice specimen extraction seems to be the optimal choice in selected cases.

## Introduction

Sigmoid volvulus (SV) rarely untwists spontaneously and typically requires urgent and effective management due to its relatively poor prognosis.^1-5^ According to current guidelines, resuscitation followed by flexible endoscopic detorsion is the first treatment choice in patients with viable sigmoid colon.^6-8^ Large-series reports support this clinical practice.^9-11^ Although the success rate of endoscopic detorsion is as high as 55%-94%, SV recurrence is not a surprise, which develops in 15%-55% of the cases.^9,11-13^ For this reason, practitioners recommend elective treatment, including various surgical or endoscopic procedures, in some selected patients. However, the selection of the process based on patient characteristics is controversial.^5,12,14-17^

In this editorial, I discuss elective treatment of SV, particularly laparoscopic sigmoid colectomy (LSC) and natural orifice specimen extraction (NOSE), based on our experience with 1076 SV cases over 57.5 years (from June 1966 to January 2024), which constitutes the most comprehensive single-center SV series worldwide.^2,4,6,9,18^

## Elective Treatment and Laparoscopic Sigmoid Colectomy in SV

Dolichosigmoid, an elongated and dilated sigmoid colon with a long mesentery, is the primary contributing factor in recurrent SV, while the other effective agents are advanced age, male gender, early SV onset, high-fiber diet habit, living in high altitude, and constipated defecation habit.^19-22^ For this reason, the basic principle in the prevention of recurrence is disfiguring the dolichosigmoid.^2,12,23^ Sigmoidopexy, sigmoidomesopexy, sigmoidomesoplasty, extraperitonealization, or percutaneous endoscopic sigmoidopexy (PES) may help in reducing the risk of recurrence.^2,5,7,8,16,23-38^ However, sigmoid colectomy, particularly LSC, is the most effective surgical technique in preventing recurrence. The mortality, morbidity, and recurrence rates associated with this procedure are 0%-2%, 10%-25%, and 0%-1%, respectively.^2,16,24,33,34,39-46^

Regarding the decision-making process in the elective treatment of SV, there are two important parameters: health status and age of the patients. In the literature, “good/bad,” “low risk/high risk,” or “uncomplicated/complicated” terms are generally used in the evaluation of general health status, while the term “young age/old age” is not standardized in the evaluation of age.^1,5,33,34,39^ As seen, such an evaluation is far from objectivity. However, the American Society of Anesthesiologists (ASA) status is an objective parameter in the evaluation of health status. Similarly, as an alternative to the age limit, “life expectancy” is a better choice, which varies from country to country and generally decreases in due course. According to this objective rating system, ASA 1-3 patients younger than the life expectancy limit are optimal candidates for elective LSC, while other alternatives, particularly PES, may be applied in cases with ASA >3 or older than the life expectancy limit.^40^

### Clinical Experience

Contrary to its low incidence in some areas, including North America, Western Europe, and Australia, SV is relatively common in Türkiye, particularly in our region, Eastern Anatolia. My colleagues and I treated 1076 SV cases over a 57.5-year period between June 1966 and January 2024. In our series, nonoperative detorsion was used in 795 patients (13 barium enema, 351 rigid endoscopy, 431 flexible endoscopy, the latter of which was used since 1988). The success rate was 83.2%, while the mortality, morbidity, and early recurrence rates were 0.6%, 2.1%, and 5.5%, respectively. Urgent surgery was preferred in 488 cases, with 17.4% mortality, 34.2% morbidity, and 0.6% early recurrence rates. Elective sigmoid colectomy (95 open and 21 laparoscopic, the latter of which was used since 2002) was applied in 116 patients. The mortality and morbidity rates were 0% and 11.2%, respectively, while no recurrence was determined in the 57 cases followed up over a mean 22.7-year follow-up period.

### Natural Orifice Specimen Extraction in SV 

According to a search of the last 79-years’ literature (between 1945 and 2024) in Web of Science^47,48^ database under the headings of “sigmoid volvulus” and “natural orifice specimen extraction,” there are only 11 publications on LSC with NOSE out of 1285 articles on SV and 427 papers on NOSE (Table 1). NOSE by using eversion, was first described by Hamada et al^49^ in 2010. Practitioners revised and upgraded trochars, anvils, staplers, and resection and extraction techniques in the later years.^50-59^ Shorter incision dimension, reduced postoperative pain, better patient comfort, earlier degassification and defecation, shorter discharge period, and better cosmetic result are major advantages of NOSE.^49-59^ However, there are still some limitations in the usage of NOSE in cases of SV:

1. About half of the cases with SV require urgent surgical management.^1,2,12,17,25,29,38,39^ This urgency prevents the usage of NOSE in most patients.
2. It was first used in 2010.^47-49^ It is still regarded as a new technique.
3. Only 7 cases were reported before 2021.^47,48,58^ Long-term outcomes remains unclear.
4. The total number of cases reported to date is 39.^47,48,55,56,58^ Healthier comments require larger patient populations.
5. There are only 11 publications, including 7 single case reports.^47-59^ Better interpretations require larger case series.
6. Different practitioners use different operative techniques, and some clinicians use robotic surgery in addition to laparoscopy.^47,48,57,58^ The techniques are not standardized.
7. The numbers, sizes, and sites of the trochars vary.^47-59^ The instruments are heterogeneous with different options available.
8. Transvaginal extraction is possible in women, unlike the common practice of the trans-anal route.^51,55,56,58,59^ The extraction ways of the specimens are variable.
9. Some surgeons split the specimen in contrast to the favorite technique, delivering the specimen intact.^51,55,56^ The extraction techniques are incompatible.
10. Almost all reports focus on adults, with only one addressing childhood SV.^49-59^ Sigmoid volvulus is not common in childhood.^60-64^ Nevertheless, its usage in children is controversial.
11. There is no pregnant patient in the reported cases.^49-59^ Although SV is uncommon in females, the disease is relatively common in pregnant women.^65-70^ The results on pregnant women are unclear.
12. Although sporadic elderly cases are present, the mean age is under 75 years in most of the reported series.^49,50,52-55,57,58^ Sigmoid volvulus is common in elderly people.^71-75^ The results on elderly individuals over 75 years old are not clear enough.
13. Body mass index is under 30 kg/m^2^ level in most patients.^55,56,58,59^ The results of its usage in morbid individuals are not bright enough.
14. Almost all cases suffers from recurrent SV.^47-59^ Its usage in primary SV is a mystery.
15. All reports involve elective or semi-elective patients.^47-59^ Its usage seems as difficult or impossible in emergency cases due to the extremely enlarged sigmoid colon.

## Conclusion

Laparoscopic sigmoid colectomy with NOSE has some nonignorable operative and postoperative advantages apart from some paramount issues. Nevertheless, it seems to be the new surgical trend in the elective treatment of SV. In my opinion, the next step may be the urgent LSC with NOSE following endoscopic or percutaneous decompression of SV.

## Figures and Tables

**Table 1.** Reports on Laparoscopic Sigmoid Colectomy with Natural Orifice Specimen Extraction in Sigmoid Volvulus

Publication	Year	Number	Age	Gender	ASA	BMI (kg/m^2^)	Trochar	Extraction	Morbidity	Discharge (day)
Hamada et al^49^	2010	1	10	M	n/a	n/a	4	A	–	n/a
Christoforidis et al^50^	2013	1	n/a	n/a	n/a	n/a	n/a	A	–	n/a
D’Hondt et al^51^	2014	1	85	F	n/a	21	SPD	V	-–	8
Wolthuis et al^52^	2015	1	n/a	F	n/a	n/a	4	V	n/a	n/a
Hsieh et al^53^	2019	2	n/a	n/a	n/a	n/a	n/a	A	–	4
Sia et al^54^	2019	1	22	M	n/a	n/a	4	A	–	n/a
Chen et al^55^	2021	16	59 (19-88)	n/a	n/a	28.2 (17-45)	4	A	n/a	5 (2-71)
Seow-En et al^56^	2021	6	68 (16-84)	3M, 3F	n/a	22.9 (21.5-23.9)	4	A	–	4 (2-9)
Devane and Martin^57^	2022	1	69	M	n/a	n/a	n/a	A	–	n/a
Uylas et al^58^	2022	8	n/a	n/a	n/a	n/a	4	A	n/a	n/a
Seow-En et al^59^	2023	1	66	M	n/a	18	3	A	–	1

A, transanal; ASA, American Society of Anesthesiologists score; BMI, body mass index; F, female; M, male; n/a, not applicable; SPD, single port device. V, transvaginal.

## References

[1] Pattanaik SK . Emergency management of sigmoid colon volvulus in a volvulus belt population and review of literature. Indian J Surg. 2018;80(6):599 605. (10.1007/s12262-017-1699-7)

[2] Atamanalp SS Peksöz R Dişçi E . Sigmoid volvulus and ileosigmoid knotting: an update. Eurasian J Med. 2022;54(suppl1):91 96. (10.5152/eurasianjmed.2022.22310) 36655451 PMC11163360

[3] Deresse T Tesfahun E Gebreegziabher ZA , et al. Perioperative adverse outcome and its predictors after emergency laparotomy among sigmoid volvulus patients: retrospective follow-up study. Open Access Emerg Med. 2023;15:383 392. (10.2147/OAEM.S430193) 37876607 PMC10591608

[4] Atamanalp SS . Sigmoid volvulus: the first one thousand-case single center series in the world. Eur J Trauma Emerg Surg. 2019;45(1):175 176. (10.1007/s00068-017-0859-6) 29079918

[5] Tian BWCA Vigutto G Tan E , et al. WSES consensus guidelines on sigmoid volvulus management. World J Emerg Surg. 2023;18(1):34. (10.1186/s13017-023-00502-x) 37189134 PMC10186802

[6] Atamanalp SS . Sigmoid volvulus. Eurasian J Med. 2010;42(3):142 147. (10.5152/eajm.2010.39) 25610145 PMC4261258

[7] Alavi K Poylin V Davids JS , et al. The American Society of Colon and Rectal Surgeons clinical practice guidelines for the management of colonic volvulus and colonic pseudo-obstruction. Dis Colon Rectum. 2021;64(9):1046 1057. (10.1097/DCR.0000000000002159) 34016826

[8] Miller AS Boyce K Box B , et al. The Association of Coloproctology of Great Britain and Ireland consensus guidelines in emergency colorectal surgery. Colorectal Dis. 2021;23(2):476 547. (10.1111/codi.15503) 33470518 PMC9291558

[9] Atamanalp SS . Endoscopic decompression of sigmoid volvulus: review of 748 patients. J Laparoendosc Adv Surg Tech A. 2022;32(7):763 767. (10.1089/lap.2021.0613) 34748412

[10] Negm S Farag A Shafiq A Moursi A Abdelghani AA . Endoscopic management of acute sigmoid volvulus in high risk surgical elderly patients: a randomized controlled trial. Langenbecks Arch Surg. 2023;408(1):338. (10.1007/s00423-023-03071-4) 37635200 PMC10460710

[11] Atamanalp SS Atamanalp RS . The role of sigmoidoscopy in the diagnosis and treatment of sigmoid volvulus. Pak J Med Sci. 2016;32(1):244 248. (10.12669/pjms.321.8410) 27022384 PMC4795878

[12] Dahiya DS Perisetti A Goyal H , et al. Endoscopic versus surgical management for colonic volvulus hospitalizations in the United States. Clin Endosc. 2023;56(3):340 352. (10.5946/ce.2022.166) 37070205 PMC10244148

[13] Atamanalp SS . Sigmoid volvulus: diagnosis in 938 patients over 45.5 years. Tech Coloproctol. 2013;17(4):419 424. (10.1007/s10151-012-0953-z) 23224856

[14] García Calonge M Muíño-Domínguez D González Sánchez MH Barreiro Alonso E . Sigmoid volvulus management, only endoscopic devolvulation? Rev Esp Enferm Dig. 2023;115(4):213 214. (10.17235/reed.2023.9488/2023) 36779459

[15] Atamanalp SS Oren D Aydinli B , et al. Elective treatment of detorsioned sigmoid volvulus. Turk J Med Sci. 2008;38:227 234.

[16] Hardy NP McEntee PD McCormick PH Mehigan BJ Larkin JO . Sigmoid volvulus: definitive surgery is safe and should be considered in all instances. Ir J Med Sci. 2022;191(3):1291 1295. (10.1007/s11845-021-02713-0) 34327621 PMC9135785

[17] Rajan R Clark DA . Current management of large bowel obstruction: a narrative review. Ann Laparosc Endosc Surg. 2022;7(23):23 23. (10.21037/ales-21-45)

[18] Atamanalp SS Disci E . Sigmoid volvulus: diagnostic modalities and sigmoid gangrene. Eurasian J Med. 2021;53(2):166 167. (10.5152/eurasianjmed.2021.21101) 34177307 PMC8184029

[19] Dişçi E Atamanalp SS . Factors precipitating volvulus formation in sigmoid volvulus. Ulus Travma Acil Cerrahi Derg. 2022;28(3):281 284. (10.14744/tjtes.2020.03762) 35485550 PMC10493538

[20] Raahave D . Dolichocolon revisited: an inborn anatomic variant with redundancies causing constipation and volvulus. World J Gastrointest Surg. 2018;10(2):6 12. (10.4240/wjgs.v10.i2.6) 29492185 PMC5827035

[21] Korkut E Peksoz R Disci E Atamanalp SS . Factors affecting recurrence in sigmoid volvulus. Pak J Med Sci. 2023;39(1):150 153. (10.12669/pjms.39.1.6882) 36694777 PMC9842996

[22] Johansson N Rosemar A Angenete E . Risk of recurrence of sigmoid volvulus: a single-centre cohort study. Colorectal Dis. 2018;20(6):529 535. (10.1111/codi.13972) 29178415

[23] Atamanalp SS Atamanalp RS . Sigmoid volvulus: avoiding recurrence. Tech Coloproctol. 2019;23(4):405 406. (10.1007/s10151-019-01984-1) 30955105

[24] Orban YA Safwat K Awad JRI Asjour H Yassin MA . Sigmoidopexy versus sigmoidectomy for sigmoid volvulus through left iliac incision in high-risk patients. Egypt J Surg. 2023;41(1):135 140.

[25] Atamanalp SS . Treatment of sigmoid volvulus: a single-center experience of 952 patients over 46.5 years. Tech Coloproctol. 2013;17(5):561 569. (10.1007/s10151-013-1019-6) 23636444

[26] Lai SH Vogel JD . Diagnosis and management of colonic volvulus. Dis Colon Rectum. 2021;64(4):375 378. (10.1097/DCR.0000000000001947) 33496483

[27] Atamanalp SS . Treatment for ileosigmoid knotting: a single-center experience of 74 patients. Tech Coloproctol. 2014;18(3):233 237. (10.1007/s10151-013-1046-3) 23839796

[28] Akhtar M Khan I . Management of viable sigmoid volvulus by mesosigmoidoplasty. Gomal J Med Sci. 2009;7(1):7 9.

[29] Atamanalp SS Disci E Peksoz R Atamanalp RS Tatar Atamanalp C . Management of sigmoid volvulus: a literature review. Ibnosina J Med Biomed Sci. 2024;16(1):5 9. (10.1055/s-0043-1776142)

[30] Bach O Rudloff U Post S . Modification of mesosigmoidoplasty for nongangrenous sigmoid volvulus. World J Surg. 2003;27(12):1329 1332. (10.1007/s00268-003-7010-z) 14595520

[31] Aharoni M Zager Y Khalilieh S , et al. Laparoscopic fixation of volvulus by extra-peritonealization: a case series. Tech Coloproctol. 2022;26(6):489 493. (10.1007/s10151-022-02596-y) 35325340

[32] Gosavi R Centauri S Teoh W Nguyen TC Narasimhan V . Laparoscopic peritoneal flap sigmoidopexy – a video vignette. Colorectal Dis. 2023;25(4):817 818. (10.1111/codi.16394) 36318596

[33] Ndong A Patel B . Safety and efficacy of laparoscopic surgery in the management of sigmoid volvulus: A systematic review and meta-analysis. Surg Open Dig Adv. 2022;6:100052. (10.1016/j.soda.2022.100052)

[34] Nguyen SH Tavares K Chinn A Russell D Gillern S Yheulon C . Is laparoscopy underutilized for sigmoid volvulus? Surg Laparosc Endosc Percutan Tech. 2022;32(5):564 570. (10.1097/SLE.0000000000001074) 35960695

[35] Frank L Moran A Beaton C . Use of percutaneous endoscopic colostomy (PEC) to treat sigmoid volvulus: a systematic review. Endosc Int Open. 2016;4(7):E737 E741. (10.1055/s-0042-106957) 27556086 PMC4993950

[36] Coron E . Should we recommend PEC and when? Endosc Int Open. 2016;4(7):E742 E743. (10.1055/s-0042-105513) 27556087 PMC4993872

[37] Jackson S Hamed MO Shabbir J . Management of sigmoid volvulus using percutaneous endoscopic colostomy. Ann R Coll Surg Engl. 2020;102(9):654 662. (10.1308/rcsann.2020.0162) 32777932 PMC7591603

[38] Farkas N Kenny R Conroy M , et al. A single centre 20-year retrospective cohort study: percutaneous endoscopic colostomy. Colorectal Dis. 2022;24(11):1390 1396. (10.1111/codi.16207) 35656558

[39] Perrot L Fohlen A Alves A Lubrano J . Management of the colonic volvulus in 2016. J Visc Surg. 2016;153(3):183 192. (10.1016/j.jviscsurg.2016.03.006) 27132752

[40] Atamanalp SS . Sigmoid volvulus: an update for Atamanalp classification. Pak J Med Sci. 2020;36(5):1137 1139. (10.12669/pjms.36.5.2320) 32704301 PMC7372645

[41] Schudrowitz N Shahan CP Moss T Scarborough JE . Bowel preparation before nonelective sigmoidectomy for sigmoid volvulus: highly beneficial but vastly underused. J Am Coll Surg. 2023;236(4):649 655. (10.1097/XCS.0000000000000593) 36695556

[42] Emna T Atef M Sarra S . Management of acute sigmoid volvulus: a Tunisian experience. Asian J Surg. 2022;45(1):148 153. (10.1016/j.asjsur.2021.04.004) 33895046

[43] Moro-Valdezate D Martín-Arévalo J Pla-Martí V , et al. Sigmoid volvulus: outcomes of treatment and predictors of morbidity and mortality. Langenbecks Arch Surg. 2022;407(3):1161 1171. (10.1007/s00423-022-02428-5) 35028738 PMC9151547

[44] Tankel J Gilshtein H Neymark M Zuckerman M Spira R Yellinek S . Sigmoidectomy following sigmoid volvulus: who is at risk of anastomotic failure? Tech Coloproctol. 2021;25(11):1225 1231. (10.1007/s10151-021-02508-6) 34480672

[45] Lee K Oh HK Cho JR , et al. Surgical management of sigmoid volvulus: a multicenter observational study. Ann Coloproctol. 2020;36(6):403 408. (10.3393/ac.2020.03.23) 33486909 PMC7837394

[46] Assenza M Ciccarone F Iannone I . Elective treatment of large-bowel obstruction in asymptomatic sigmoid volvulus. Clin Ter. 2021;171(6):e466 e470. 10.7417/CT.2020.2258 33151242

[47] Web of Science. Sigmoid volvulus. Available at: https://www.wwebofscience.com/wos/woscc/summary/1580485c-b7b8-4963-bc42-d09d52736d37-c151c905/relevance/1. Accessed January 26, 2024.

[48] Web of Science. Natural orifice specimen extraction. Available at: https://www.webofscience.com/wos/woscc/summary/81c9a880-d273-4012-80b8-78c74c82907b-a6c381b4/relevance/1. Accessed January 26, 2024.

[49] Hamada T Hirose R Kosaka T Fujita F Tajima Y Kanematsu T . Laparoscopic sigmoidectomy using a prolapsing technique for sigmoid colon volvulus in children. Eur J Pediatr Surg. 2010;20(1):50 52. (10.1055/s-0029-1192049) 19370518

[50] Christoforidis D Clerc D Demartines N . Transrectal specimen extraction after laparoscopic left colectomy: a case-matched study. Colorectal Dis. 2013;15(3):347 353. (10.1111/codi.12006) 23030665

[51] D’Hondt M Devriendt D Van Rooy F Vansteenkiste F Dozois E . Transvaginal pure NOTES sigmoid resection using a single port device. Tech Coloproctol. 2014;18(1):77 80. (10.1007/s10151-013-1005-z) 23564271

[52] Wolthuis AM de Buck van Overstraeten A Fieuws S Boon K D’Hoore A . Standardized laparoscopic NOSE-colectomy is feasible with low morbidity. Surg Endosc. 2015;29(5):1167 1173. (10.1007/s00464-014-3784-3) 25149636

[53] Hsieh YC Lee JY Chen YC Hsieh JS . Elective laparoscopic resection for sigmoid volvulus with natural orifice specimen extraction. Videoscopy. 2019;29(1). (10.1089/vor.2018.0549)

[54] Sia TC Cartmill J Keshava A Gilmore A . Natural orifice specimen extraction for high anterior resection – technical tips including an original and effective technique for atraumatic specimen extraction – a video vignette. Colorectal Dis. 2019;21(7):849 850. (10.1111/codi.14640) 30980455

[55] Chen MZ Cartmill J Gilmore A . Natural orifice specimen extraction for colorectal surgery: early adoption in a Western population. Colorectal Dis. 2021;23(4):937 943. (10.1111/codi.15455) 33226716

[56] Seow-En I Chang SC Ke TW Shen MY Chen HC Chen WTL . Uncomplicated sigmoid volvulus is ideal for laparoscopic sigmoidectomy with transrectal natural orifice specimen extraction. Dis Colon Rectum. 2021;64(5):e90 e93. (10.1097/DCR.0000000000001922) 33496476

[57] Devane LA Martin ST . Robotic sigmoid colectomy with transanal extraction – a video vignette. Colorectal Dis. 2022;24(1):147 148. (10.1111/codi.15932) 34605150

[58] Uylas U Gunes O Kaplan K . A review of sigmoid volvulus and natural orifice specimen extraction surgery. Ann Laparosc Endosc Surg. 2022;7:25. (10.21037/ales-22-16)

[59] Seow-En I Li KK Khor SN Tan EKW . Laparoscopic sigmoid colectomy with transanal natural orifice specimen extraction for sigmoid volvulus – A video vignette. Colorectal Dis. 2023;25(8):1746 1747. (10.1111/codi.16671) 37469137

[60] Damkjaer MB Farooqui W Ifaoui I Penninga L . Sigmoid volvulus in children. BMJ Case Rep. 2021;14(5)(suppl2):492 495. (10.1136/bcr-2021-241869) PMC8117995 33980558

[61] Lee B Wu A . Pediatric sigmoid volvulus. Pediatr Emerg Care. 2019;35(12):e232 e233. (10.1097/PEC.0000000000001470) 29596283

[62] Atamanalp SS Yildirgan MI Başoğlu M Kantarci M Yilmaz I . Sigmoid colon volvulus in children: review of 19 cases. Pediatr Surg Int. 2004;20(7):492 495. (10.1007/s00383-004-1222-7) 15241618

[63] Parolini F Orizio P Bulotta AL , et al. Endoscopic management of sigmoid volvulus in children. World J Gastrointest Endosc. 2016;8(12):439 443. (10.4253/wjge.v8.i12.439) 27358669 PMC4919692

[64] Martin G Montalva L Paré S , et al. Robotic assisted colectomy in children: a comparative study with laparoscopic surgery. J Robot Surg. 2023;17(5):2287 2295. (10.1007/s11701-023-01647-2) 37336840

[65] Bajaj M Gillespie C Dale J . Recurrent sigmoid volvulus in pregnancy. ANZ J Surg. 2017;87(11):E226 E227. (10.1111/ans.13140) 25913147

[66] Atamanalp SS Öztürk G . Sigmoid volvulus in pregnancy. Turk J Med Sci. 2012;42(4):9 15. (10.3906/sag-1101-2)

[67] Cortez N Berzosa M Muddasani K Ben-David K . Endoscopic decompression of sigmoid volvulus in pregnancy. J Investig Med High Impact Case Rep. 2020;8:1 3. 10.1177/2324709620975939 PMC7705807 33238755

[68] Mohamed I Hassan N Fatima I , et al. Management of recurrent sigmoid volvulus in pregnancy: a case report. Am J Gastroenterol. 2023;118(10):2509.

[69] Wang MY Zheng A Li JK . Laparoscopic treatment of mesosigmoid adhesion-caused spontaneous sigmoid volvulus in the second trimester of pregnancy. Asian J Surg. 2023;46(1):611 612. (10.1016/j.asjsur.2022.07.007) 35868961

[70] Nada AA Gergi P . Sigmoid volvulus in a pregnant female: case presentation. Egypt J Surg. 2022;41(1):463 468.

[71] Avots-Avotins KV Waugh DE . Colon volvulus and the geriatric patient. Surg Clin North Am. 1982;62(2):249 260. (10.1016/s0039-6109(16)42684-8) 7071692

[72] Dolejs SC Guzman MJ Fajardo AD Holcomb BK Robb BW Waters JA . Contemporary management of sigmoid volvulus. J Gastrointest Surg. 2018;22(8):1404 1411. (10.1007/s11605-018-3747-4) 29569006

[73] Atamanalp SS Ozturk G . Sigmoid volvulus in the elderly: outcomes of a 43-year, 453-patient experience. Surg Today. 2011;41(4):514 519. (10.1007/s00595-010-4317-x) 21431484

[74] Rajsiddharth B Patlolla SR Reddy BS Sriramoju S Palley BK Maripeddi K . A clinical study of sigmoid volvulus. Int J Scient Study. 2016;3(10):158 162.

[75] Bruzzi M Lefèvre JH Desaint B , et al. Management of acute sigmoid volvulus: short- and long-term results. Colorectal Dis. 2015;17(10):922 928. (10.1111/codi.12959) 25808350

